# Transanal ileal pouch-anal anastomosis: A systematic review and meta-analysis of technical approaches and clinical outcomes

**DOI:** 10.1007/s00423-024-03343-7

**Published:** 2024-05-06

**Authors:** Ian J. B. Stephens, Kevin G. Byrnes, John P. Burke

**Affiliations:** 1 Department of Colorectal Surgery, Beaumont Hospital, Dublin 9, Ireland; 2 School of Pharmacy and Biomolecular Sciences, Royal College of Surgeons Ireland, 123 St. Stephens Green, Dublin, Ireland; 3 Havering and Redbridge University Trust, Greater London, UK

**Keywords:** Transanal, Ileoanal, Ulcerative Colitis, Systematic Review, Meta-analysis

## Abstract

**Purpose:**

Transanal minimally invasive surgery has theoretical advantages for ileal pouch-anal anastomosis surgery. We performed a systematic review assessing technical approaches to transanal IPAA (Ta-IPAA) and meta-analysis comparing outcomes to transabdominal (abd-IPAA) approaches.

**Methods:**

Three databases were searched for articles investigating Ta-IPAA outcomes. Primary outcome was anastomotic leak rate. Secondary outcomes included conversion rate, post operative morbidity, and length of stay (LoS). Staging, plane of dissection, anastomosis, extraction site, operative time, and functional outcomes were also assessed.

**Results:**

Searches identified 13 studies with 404 unique Ta-IPAA and 563 abd-IPAA patients. Anastomotic leak rates were 6.3% and 8.4% (RD 0, 95% CI -0.066 to 0.065, p = 0.989) and conversion rates 2.5% and 12.5% (RD -0.106, 95% CI -0.155 to -0.057, p = 0.104) for Ta-IPAA and abd-IPAA. Average LoS was one day shorter (MD -1, 95% CI -1.876 to 0.302, p = 0.007). A three-stage approach was most common (47.6%), operative time was 261(± 60) mins, and total mesorectal excision and close rectal dissection were equally used (49.5% vs 50.5%). Functional outcomes were similar. Lack of randomised control trials, case-matched series, and significant study heterogeneity limited analysis, resulting in low to very low certainty of evidence.

**Conclusions:**

Analysis demonstrated the feasibility and safety of Ta-IPAA with reduced LoS, trend towards less conversions, and comparable anastomotic leak rates and post operative morbidity. Though results are encouraging, they need to be interpreted with heterogeneity and selection bias in mind. Robust randomised clinical trials are warranted to adequately compare ta-IPAA to transabdominal approaches.

**Supplementary Information:**

The online version contains supplementary material available at 10.1007/s00423-024-03343-7.

## Introduction

Restorative proctocolectomy with ileal pouch-anal anastomosis (IPAA) provides a means of re-establishing intestinal continuity after resection of the colon and rectum[1]. Ulcerative disease and familial adenomatous polyposis (FAP) are the most common indications for proctocolectomy [2–4]. These patients are a relatively young cohort, with the majority undergoing surgery between the 4th-6th decade of life[5–7]. Despite risks of early and late complications, quality of life improves after restorative proctocolectomy in ulcerative colitis [8] and pouch function remains robust over time[6]. Early and late operative outcomes such as pelvic sepsis and anastomotic stricture are independent predictors of pouch failure[9].

Laparoscopic IPAA was introduced 1990s and has shorter hospitalisation, better cosmetic result and improved female fecundity when compared to open surgery[10, 11]. More recently robotic platforms, single incision approaches, and natural orifice specimen extraction have been described and shown to be feasible and safe[12–15]. Transanal surgery offers a unique anatomical perspective to the pelvis. Its application in IPAA was described in 2015 in cadaveric[16] and animal models[17], and then in clinical practice[15, 18, 19] A transanal laparoscopic port is inserted into the anal canal. A purse-string suture is used to close the rectal lumen 3-4 cm above the dentate line, a full thickness rectotomy is then performed circumferentially 1 cm distal to this. Dissection of the distal rectum is continued either in the total mesorectal excision (TME) or close rectal dissection (CRD) plane until it meets the abdominal dissection [20]. The approach provides an alternative means of dividing the distal rectum and performing the stapled anastomosis when compared to the often-challenging cross-stapling of the rectum and creation of the ileoanal anastomosis in the deep pelvis from above.

The technical benefits of Ta-IPAA are 1) direct visualisation of the rectal mucosa to ensure optimum cuff length, 2) single-staple firing, 3) ease of access to the narrow bony pelvis, and 4) two team simultaneous approach, however the significant learning curve of transanal surgery must be considered[20]. If these benefits translate meaningfully into clinical practice, they may result in lower anastomotic leak rates, reduced need for conversion to open surgery, shorter operative time, and better functional outcomes.

This study aims to systematically review the literature on Ta-IPAA to investigate technical characteristics and clinical and functional outcomes after Ta-IPAA and perform a meta-analysis comparing short-term clinical outcomes to established transabdominal (abd-IPAA) approaches.

## Materials Methods

### Search Strategy

Systematic review and meta-analysis of the literature was performed in accordance with the Preferred Reporting Items for Systematic Review and Meta-Analysis (PRISMA) 2020 statement [21] to identify studies reporting on transanal IPAA outcomes. On March 22nd, 2023 a search of EMBASE, Pubmed, and Cochrane Library databases was performed. The search terms “laparoscopic”, “transabdominal”, “open”, “minimally invasive”, “tapouch” were used in combination with “ipaa”, “tapouch”, “j pouch”, “ileoanal” or “restorative proctocolectomy”. To be eligible for inclusion articles had to be published in English language in peer-reviewed publications. All indications for IPAA were eligible for inclusion. Published abstracts, presentations, case reports, and studies reporting exclusively paediatric cohorts were excluded. Data was accrued and stored in a password protected Microsoft Excel Data Sheet, using a predefined template. Author name, country, year of publication, journal, study design and patient number were extracted for each article. Where applicable operative approach, number of stages, technical characteristics, operative duration, post operative morbidity, anastomotic leak, length of hospital stay (LoS), conversion rates, and functional outcomes were extracted and collected. Number of stages were defined as either single stage (restorative proctectomy without covering ileostomy), 2-stage (restorative proctocolectomy with loop ileostomy followed by reversal of ileostomy), modified 2-stage (Total abdominal colectomy with end ileostomy followed by proctectomy with IPAA formation without covering ileostomy), or 3-stage (total abdominal colectomy with end ileostomy, followed by proctectomy with IPAA formation with loop ileostomy, and finally reversal of loop ileostomy [22]. The study protocol was published on Open Science Foundation Registry (DOI 10.17605/OSF.IO/JAD4S) and registered on the PROSPERO database (ref: CRD42023418322) [23].

### Outcome Measures

Primary outcome was anastomotic leak defined as a defect of the intestinal wall integrity at the pouch-anal anastomotic site leading to a communication between the intra and extraluminal compartments, including a perianastomotic abscess, as diagnosed by radiological investigation, reoperation, or examination of the neorectum under anaesthesia or at endoscopy [24]. Secondary outcomes included operative time, LoS, post operative morbidity, and conversion rates. Descriptive outcomes included quality of life and functional indicators. Comparative studies were considered eligible for meta-analysis if they reported on outcomes following Ta-IPAA and abd-IPAA (open, single, or multi-port laparoscopic, and/or robotic).

### Study Overlap

Study overlap was addressed on a case-by-case basis for each outcome measure. The cohorts and study period in each article were carefully examined and partial and complete overlaps identified. These were represented graphically by Venn diagram. For a given outcome, the overall aggregated study populations were examined, and papers excluded in a fashion to minimise overall case loss while avoiding double counting. In some cases, this was straightforward while in others it required an examination of all possible inclusion permutations to ensure minimal losses.

### Statistical Analysis

Extracted data was transferred into Review Manager 5.4.1 software (The Cochrane Collaboration, 2020) [25] for meta-analysis. Graphs were created using StataSE® 16 [26]. Continuous variables were standardised to mean ± standard deviation using Hozo [27] and Luo and Wan [28, 29] methods. For continuous variables (operative time, LoS) the mean difference and 95% confidence intervals (CI) were calculated. A DerSimonian and Laird method inverse-variance random-effects model was used to determine pooled outcome measures [30]. Heterogeneity between studies was calculated by the inconsistency test (I^2^). For dichotomous variables (anastomotic leak, conversion rate, morbidity), risk difference (RD) was analysed with its variance and 95% confidence interval. GRADEpro GDT was used to assess certainty of evidence [31]. Statistical analysis was performed by IS and KB.

## Results

### Study Characteristics

**Fig. 1:**
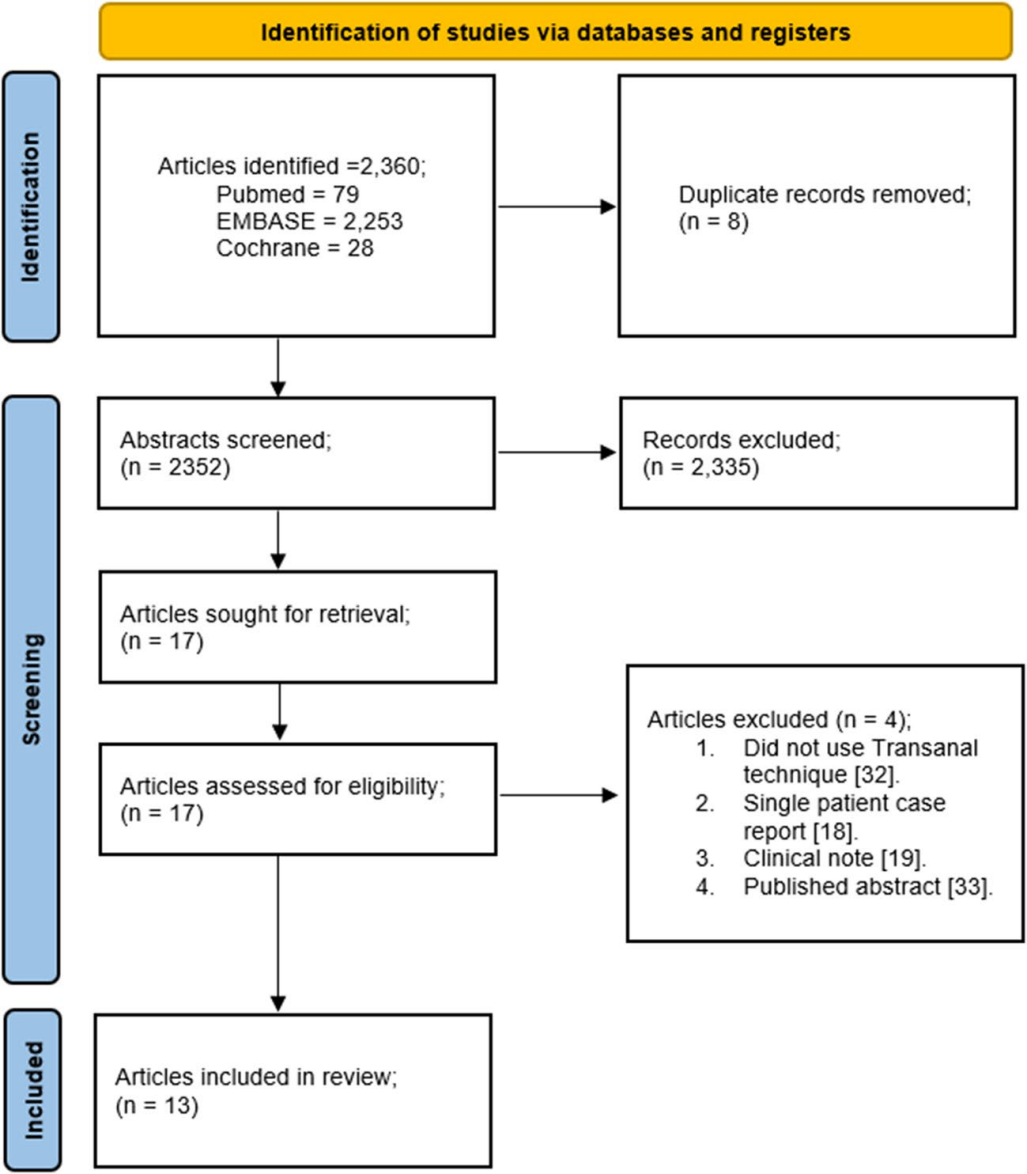
Prisma flowchart of search strategy

A total of 2,360 articles were identified for consideration. Of these, 2,253 were from EMBASE, 79 from Pubmed, and 28 from Cochrane library. There were 8 duplicates removed, and 2,352 abstracts were screened. A total of 17 papers were taken forward for full assessment, and of these 4 were excluded [18, 19, 32, 33] (Fig. 1).

**Fig. 2:**
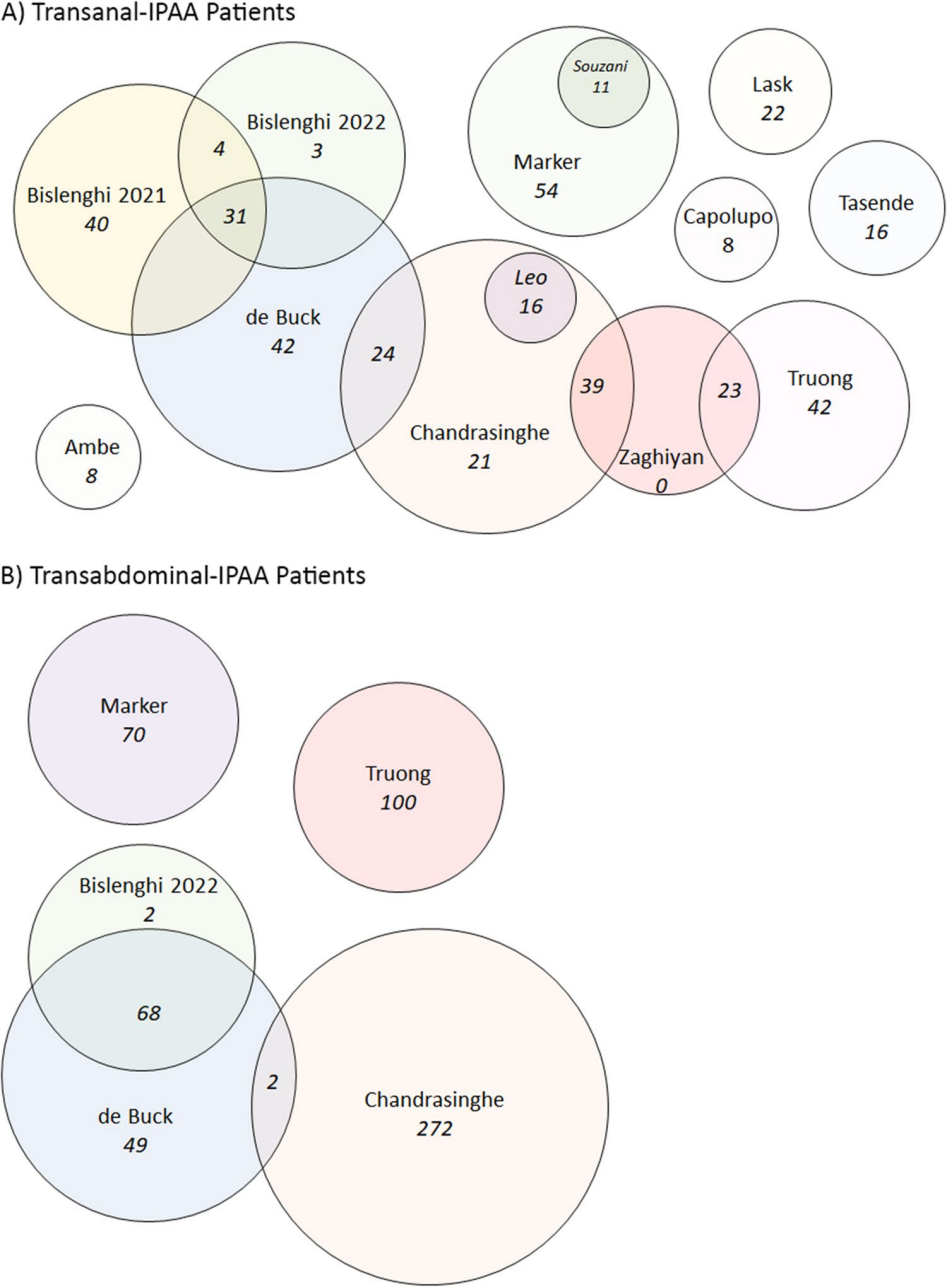
Overlap Analysis. Venn diagrams demonstrating (**A**) Patient overlap for transanal patients between studies, (**B**) Patient overlap for transabdominal patients. 179 patients overlapped in the Ta-IPAA group, and 70 in the abd-IPAA cohorts, leaving a total of 404 unique Ta-IPAA patients, and 563 abd-IPAA patients across the studies

Of the 13 papers identified, all were published after 2015. Of these, five were small (8–16 patients) single centre case series, five were single centre cohort or comparative studies (22–65 Ta-IPAA patients), and three were large multicentre studies (62–100 Ta-IPAA patients) (Table 1). There were no randomised control trials. A gross total of 583 Ta-IPAA and 633 abd-IPAA patients were included, however 179 Ta-IPAA and 70 abd-IPAA patients appeared in two or more studies. This resulted in a total of 404 unique Ta-IPAA and 563 abd-IPAA after overlapping cases were considered (Fig. 2). All patients from Souzani 2019 and Leo 2016 were included in Marker 2022 and Chandrasinghe 2020 respectively, which resulted in their exclusion from analysis unless the outcome of interest was absent from the larger studies. The other overlaps were more complex and resulted in cascading exclusions. For example, when an outcome was present in all studies exclusion of de Buck 2017, Leo 2016, Bislenghi 2022, Souzani 2019, and Zaghiyan 2018 resulted in the minimum patient loss (45) as the remaining patients from these studies were captured in Chandrasinghe 2020, Truong 2022, Marker 2022, and Bislenghi 2021 giving a total of 359 patients for analysis (supplemental information 1).

**Table 1:** Summary of included articles

Author	Country	Publication Year	Study Design	Patient Numbers	Patient Age (mean ± SD)	Journal	Aim
Tasende et al. [15]	Spain	2015	Single centre, case series	16	40.5 ± 15.7	Surgical Endoscopy	Initial experience with Ta-IPAA
Leo et al. [34]	UK	2016	Single centre, case series	16	46 ± 11	Colorectal Disease	Technical description, Ta-IPAA combined with Single-incision abdominal approach
Ambe et al. [35]	Germany	2017	Single centre, case series	8	20.5 ± 4.6	Techniques in Coloproctology	Assess outcomes after Ta-IPAA in FAP
de Buck et al. [36]	Belgium, Netherlands, Denmark	2017	Multicentre, comparative cohorts	219 Total 97 Transanal 119 Transabdominal	Transanal 37 ± 17.8 Transabdominal 39 ± 13.3	Annals of Surgery	Compare outcomes and technical features of Ta-IPAA and Abd-IPAA
Zaghiyan et al. [37]	US, UK, Sri Lanka	2018	Multicentre, comparing technical approaches	62	38 ± 13	Techniques in Coloproctology	Assess technical variations in application, and feasibility of Ta-IPAA in IBD internationally
Souzani et al. [38]	Denmark	2019	Single centre, case series	11	31 ± 12	Asian Journal of Endoscopic Surgery	Initial experience with Ta-IPAA
Bislenghi et al. [39]	Belgium	2021	Single centre, cohort study	75	34 ± 9	Colorectal Disease	Short term outcomes of Ta-IPAA following modified two-stage approach
Chandrasinghe et al. [40]	UK, Netherlands, Italy	2020	Multicentre, retrospective comparative cohort	374 Total 100 Transanal 274 Transabdominal	Transanal 39.9 ± 12.76 Transabdominal 38.2 ± 13.2	Journal of Crohn's and Colitis	Compare long-term functional outcomes between Ta-IPAA and abd-IPAA
Capolupo et al. [41]	Italy	2021	Single centre, case series	8	53.75 ± 14.7	BMC Surgery	Initial experience with Ta-IPAA
Lask et al. [42]	Germany	2021	Single centre, retrospective cohort	22	32 ± 12.5	Patient Safety in Surgery	Determine anastomotic leak rates post Ta-IPAA as well as short + long term pouch function
Marker et al. [43]	Denmark	2022	Singe centre, retrospective comparative cohort	135 Total 65 Transanal 70 Transabdominal	Transanal 32 ± 12 Transabdominal 30 ± 12	Techniques in Coloproctology	Compare short term outcomes between Ta-IPAA and Lap-IPAA
Bislenghi et al. [44]	Belgium	2022	Single centre, retrospective comparative cohort	108 Total 38 Transanal 70 Transabdominal	Transanal 36.7 ± 14.8 Transabdominal 39.1 ± 13.3	Langenbeck's Archives of Surgery	Compare functional outcomes and QoL after Ta-IPAA or abd-IPAA for UC
Truong et al. [45]	US	2022	Single centre, prospective comparative cohort	165 Total 65 Transanal 100 Transabdominal	Transanal 37 ± 15 Transabdominal 37.8 ± 18	Diseases of Colon and Rectum	Initial experience with Ta-IPAA, short term clinical outcomes

QOL = Quality of Life. UC = Ulcerative Colitis. FAP = Familial adenomatous polyposis

### Technical Aspects and Clinical Outcomes of Ta-IPAA

All 13 papers reported on indication, number of stages, use of close rectal dissection (CRD) or total mesorectal excision (TME), and type of anastomosis. 9 reported on the number of teams used and conversion rates. Eleven papers reported on operative time and extraction site (Table 2). The use of covering ileostomy can be inferred by operative stages, de Buck et al. (3.1% 1-stage, 52.6% modified 2-stage), Bislenghi et al. 2021 and 2022 (100%, 92.11% modified 2-stage), and Chandrasinghe et al. 2022 (54% modified 2-stage) used well studied temporary ileostomy omitting approaches. Ambe et al. 2017 use a “ghost ileostomy”, which has been described elsewhere [46], as an alternative to traditional ostomy formation.

**Table 2:** Summary of Ta-IPAA operative characteristics for all studies

Article	Patient No	Indication	No. of Stages	TME/CRD	1 or 2 Team Approach	Operative Time (mins—mean ± SD)	Conversion Rate (event no.)	Extraction Site	Anastomosis
Tasende et al. 2015	16	Ulcerative Colitis	3-stage	CRD	2	170 ± 50	0% (0)	Transanal	Stapled—87.5% Handsewn -12.5%
Leo et al. 2016	16	Ulcerative Colitis	2-stage—18.75% 3-stage—81.25%	TME	2	247 ± 71	18.75% (3)	Stoma site	Stapled—87.5% Handsewn—12.5%
Ambe et al. 2017	8	FAP	1-stage (with "ghost ileostomy")	TME	1	512 ± 104	0% (0)	Suprapubic	Stapled
de Buck et al. 2017	97	Ulcerative Colitis	1-stage—3.1% 2-stage—4.1% Modified 2-stage—52.6% 3-stage—40.2%	TME—45.4% CRD—54.6%	not specified	211 ± 54	5.2% (5)	Stoma site—7.2%, Transanal—42.3%, Pfannenstiel—20.6% Laparotomy—2.1% Umbilical—1%, Unspecified—26.8%	Stapled—97.9% Handsewn—2.1%
Zaghiyan et al. 2018	62	Ulcerative Colitis (97%) Indeterminate Colitis (3%)	2-stage—11% 3-stage—89%	TME—79% CRD—21%	predominantly 2 team	266 ± 99	not reported	Stoma site—56%, Transanal—42%	Stapled—81% Handsewn—19%
Souzani et al. 2019	11	Ulcerative Colitis	3-stage	CRD	1	285 ± 52	0% (0)	Transanal	Stapled
Bislenghi et al. 2021	75	Ulcerative Colitis	Modified 2 stage	TME—2.7% CRD—97.3%	not specified	159 ± 23	4% (3)	Transanal—88%, Stoma site—12%	Stapled
Chandrasinghe et al. 2020	100	Ulcerative Colitis	1-stage—1% 2-stage—11% modified 2-stage—54% 3-stage—34%	TME—41.6% CRD—58.3%	2	not reported	not reported	not reported	Stapled
Capolupo et al. 2021	8	Ulcerative Colitis	2 -stage—25% 3-stage—75%	CRD	2	326 ± 69	0% (0)	Pfannenstiel—87.5% Stoma—12.5%	Stapled
Lask et al. 2021	22	Ulcerative Colitis	2-stage—13.6% 3-stage—86.4%	TME	2	362 ± 163	not reported	Port site (single port abdominal phase)	Stapled—36.3% Handsewn—63.6%
Marker et al. 2022	65	Ulcerative Colitis	3-stage	CRD	not specified	277 ± 55	0% (0)	^1^Stoma site—74% Midline—3% Suprapubic—23%	Stapled
Bislenghi et al. 2022	38	Ulcerative Colitis	Modified 2-stage—92.11% 3-stage—7.89%	TME—2.7% CRD—97.3%	not specified	not reported	0% (0)	not reported	Stapled—97.4% Handsewn—2.6%
Truong et al. 2022	65	Ulcerative Colitis (83%) Indeterminate colitis (11%) Crohn’s Disease (6%)	2-stage – 11% 3-stage – 89%	TME	2	256 ± 45	not reported	Stoma site preferred	^2^Handsewn—71% Stapled—29%

CRD = Close Rectal Dissection, TME = Total mesorectal excision. 1 – Marker et al., report site of pouch creation in lieu of specimen extraction site. 2 – These are rates of anastomosis type across all 165 patients, not on Ta−IPAA individually as this is not included in Truong et al.

Ulcerative colitis was the most common indication, accounting for 94.7% of cases. Modified 2-stage (43.9%) and 3-stage (47.6%) were the most employed operative strategies, and CRD (50.5%) and TME (49.5%) were equally used. The stoma site (37%) was the most used extraction site, followed by transanal (28%). Weighted mean operative time was 261 ± 60 min.

The definition of an anastomotic leak varied between papers. Truong 2022, Bislenghi 2020, de Buck 2017, and Marker 2022 included both clinical and radiological leaks defined as identified either by radiological assessment demonstrating contrast enema extravasation at, or a defect in the anastomosis with or without perianastomotic fluid or abscess, or clinically at time of reoperation or rectal examination under anaesthesia, whereas Zaghiyan 2018, Chandrasinghe 2019, and Souzani 2019 also included perianastomotic pelvic abscesses without communication. Lask 2021 exclusively made the diagnosis by endoscopy with evidence of anastomotic defect. The remaining papers did not define anastomotic leakage, with Ambe 2017, Tasende 2015, and Capolupo reporting no leaks.

**Fig. 3:**
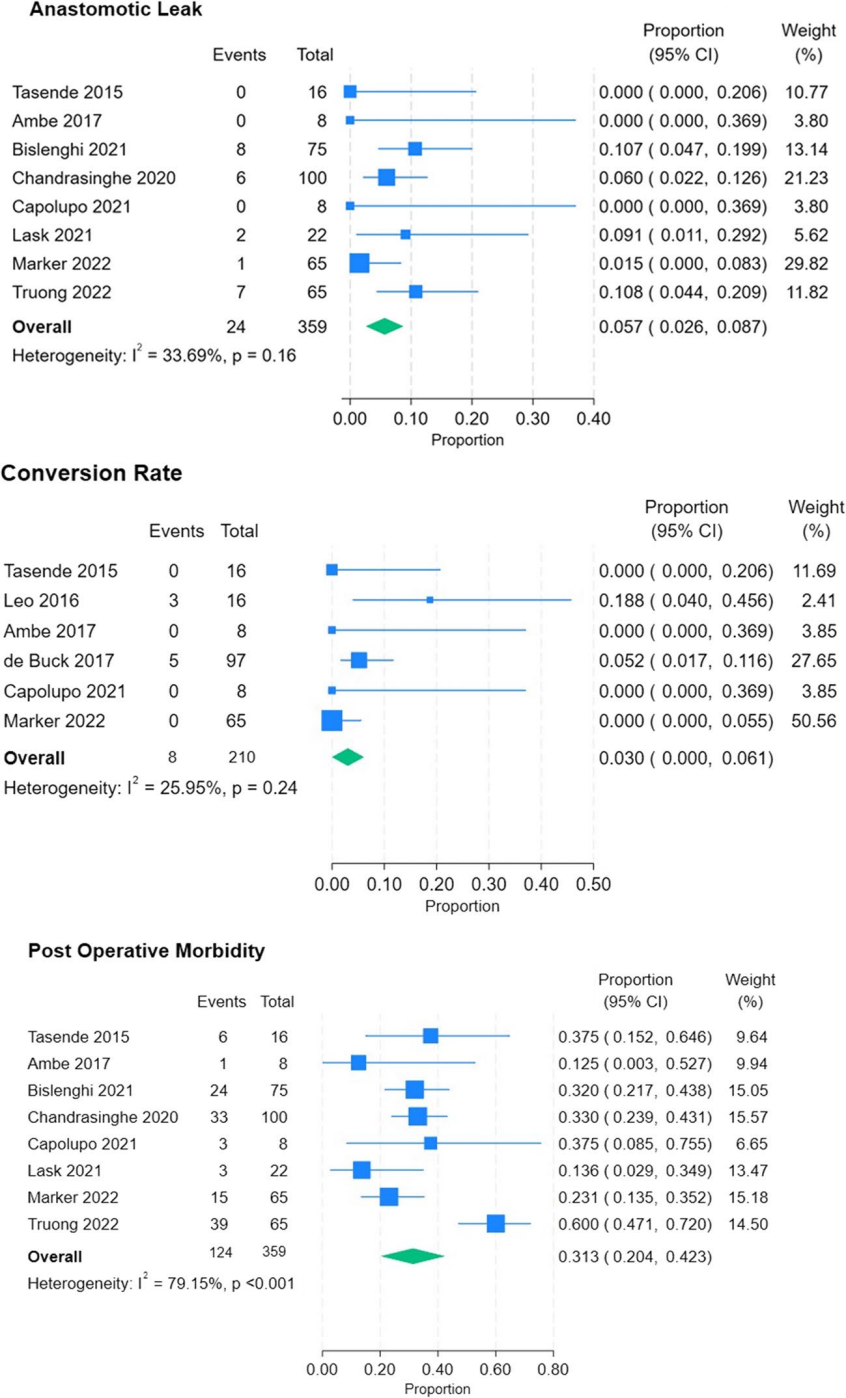
Non-comparative outcomes forest plots. Proportion of events as fraction of total population is plotted for each variable. I^2^ describes study heterogeneity, and p values relate to significance of heterogeneity

For non-comparative analysis, anastomotic leak rate after Ta-IPAA was 5.7% (CI 2.6–8.7%), conversion rate 3% (CI 0–0.06%), and post operative morbidity 31.3% (CI 20.4–42.3%). Study heterogeneity varied between variables assessed, I^2^ was 33.69% (p = 0.16), 25.95% (p = 0.24), and 79.15% (p < 0.001) respectively (Fig. 3). Weighted mean LoS was 6.7 days (± 2).

### Comparative Outcomes

Five studies included comparative Ta-IPAA and abd-IPAA cohorts. Bislenghi 2022, Chandrasinghe, and de Buck include a variety of abd-IPAA techniques (open, single/multiple port laparoscopy, and/or robotic), whereas Truong is an exclusively open cohort, and Marker is exclusively laparoscopic. Not all studies report conversion rates or LoS, and de Buck et al. reports anastomotic leak rate across its total cohort, without a breakdown of Ta-IPAA and abd-IPAA events.

**Fig. 4:**
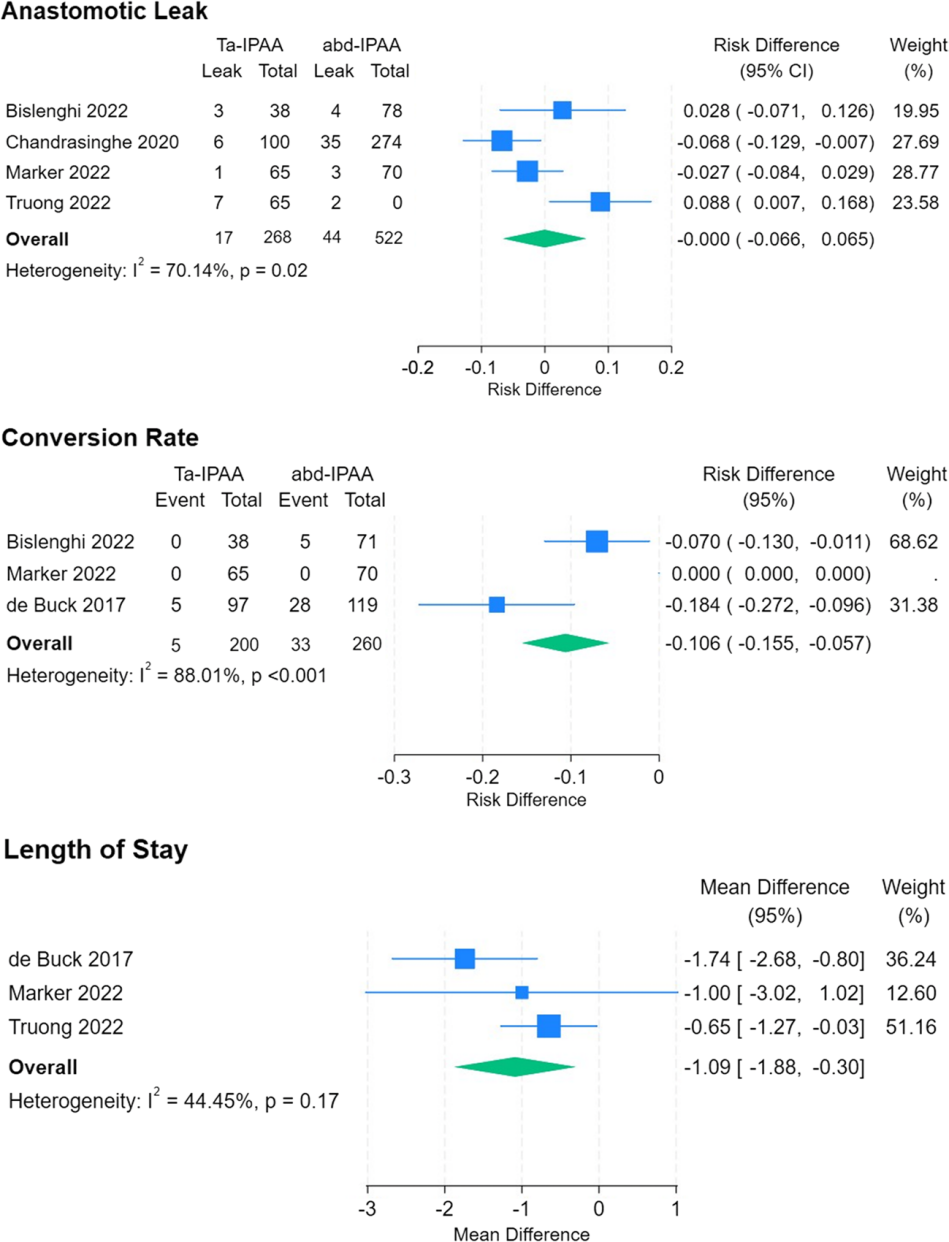
Meta-analysis of Ta-IPAA compared to abd-IPAA. Left side of plot favours Ta-IPAA, right side favours abd-IPAA. Risk difference is reported for dichotomous variables, and mean difference for continuous variables. I^2^ describes study heterogeneity, and p values relates to the significance of heterogeneity

Weighted mean age for Ta-IPAA was 36.7(± 4.15), and 37.5(± 2.6) years for abd-IPAA. There was no significant difference in risk (RD 0, CI -0.066 to 0.065, p = 0.989) of anastomotic leak rate (6.3% Ta-IPAA, 8.4% abd-IPAA), or conversion rate (2.5% Ta-IPAA, 12.7% abd-IPAA, RD -0.106, 95% CI -0.155 to -0.057, p = 0.104) between approaches. Study heterogeneity (I^2^) was significant in anastomotic leak comparison (70.14%, p = 0.02) and conversion rate analysis (88%, p < 0.001). LoS was one day shorter with Ta-IPAA compared to abd-IPAA (mean difference -1.09, CI -1.88 to -0.3, p = 0.007). Study heterogeneity did not affect analysis (44.44%, p = 0.17) (Fig. 4).

**Fig. 5:**
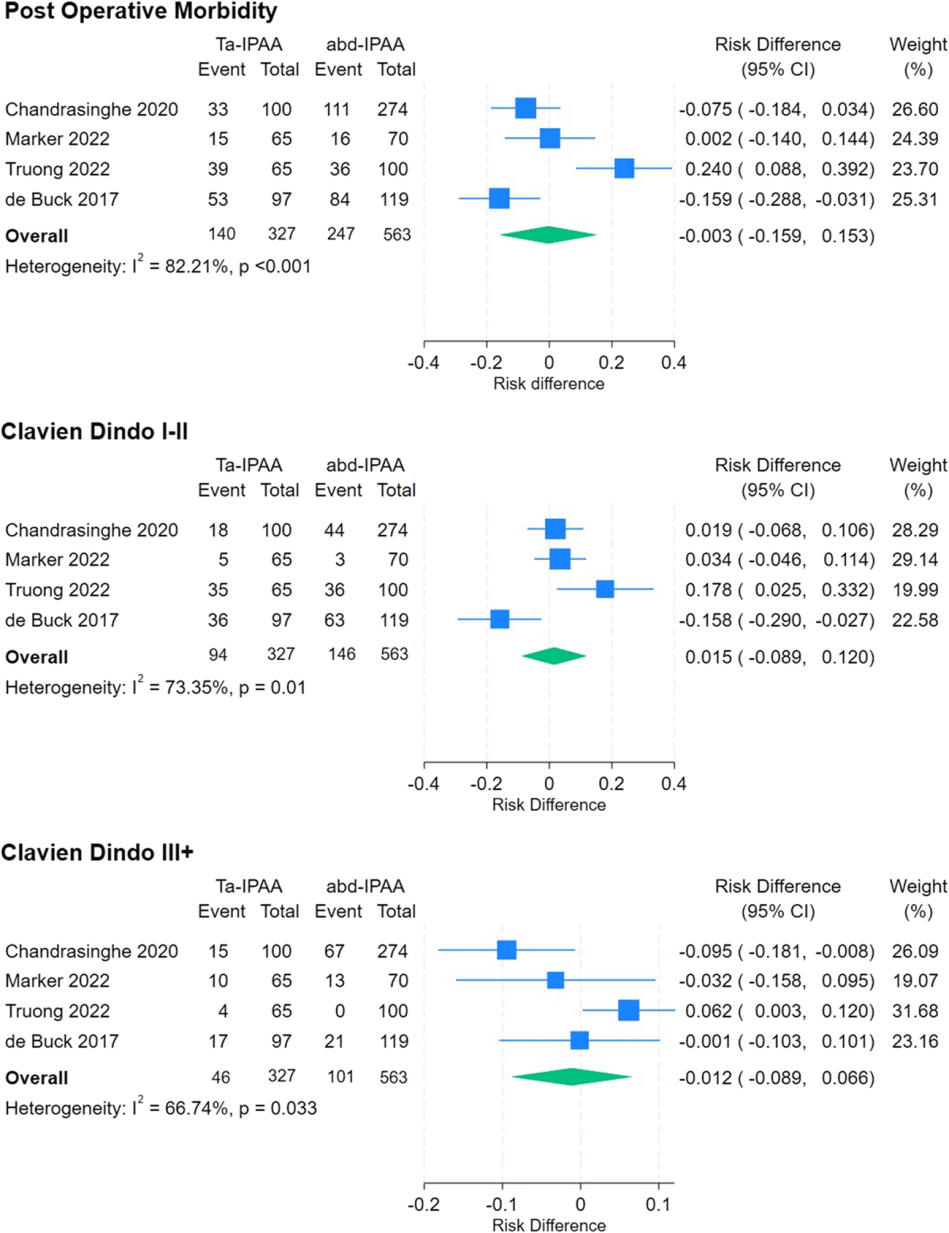
Meta-analysis of post operative co-morbidity of Ta-IPAA compared to abd-IPAA. Left side of plot favours Ta-IPAA, right side favours abd-IPAA. I^2^ describes study heterogeneity, and p values relates to the significance of heterogeneity

Overall post operative morbidity was comparable between approaches (42.8% Ta-IPAA, 43.9% abd-IPAA, RD -0.003, CI -0.159 to 0.153, p = 0.971). As were rates of Clavien Dindo Class I-II (28.7% Ta-IPAA, 25.9% abd-IPAA, RD 0.015, CI -0.089 to 0.120, p = 0.773), and Class III + (14.1% Ta-IPAA, 17.9% abd-IPAA, RD -0.012, CI -0.089 to 0.066, p = 0.771) complications. There was significant study heterogeneity across morbidity analysis (overall 82.21%, p < 0.001, Clavien Dindo I-II 73.35%, p = 0.010, Clavien Dindo III + 66.74%, p = 0.033) (Fig. 5). There is a small overlap between de Buck and Chandrasinghe (24 ta-IPAA, 2 abd-IPAA). In subgroup analysis with exclusion of de Buck on this basis, the outcomes of comparative meta-analysis (morbidity RD -0.05, CI -0.131 to 0.231, p = 0.537, Clavien Dindo I-II RD 0.055, CI -0.02 to 0.129, p = 0.148, and Clavien Dindo III + RD -0.017, CI -0.124 to 0.090, p = 0.755) still do not reach significance and was comparable to the full analysis and study heterogeneity remains high (I^2^ = 81.9% p < 0.001, I^2^ = 39.5% p = 0.19, I^2^ = 77.8% p = 0.01). Certainty of evidence as per GRADEpro GDT online assessment tool was very low for anastomotic leak, conversion rate, and post operative morbidity, and low for length of stay.

### Functional outcomes

Two studies compared functional outcomes after transanal and transabdominal IPAA. Chandrasinghe 2020 followed patients at 6 weeks, 3, 6, and 12 months post operatively. Mean Cleveland Global Quality of Life score was similar between Ta-IPAA and abd-IPAA (0.75 ± 0.12, 0.71 ± 14; p = 0.11), but the transanal approach scored better on quality of health (7.30 vs 7.73, p = 0.04) and energy level (6.68 vs 7.17, p = 0.03). 24-h stool frequency was comparable between groups, with similar portions of patients reporting 10 or less stools (Ta-IPAA 78%, abd-IPAA 79%; p = 0.77).

Bislenghi 2022 similarly followed patients at 1, 3, 6, and 12 months. Global Quality of Life scale (GQOL) [47] was higher in transanal group compared to transabdominal (82.7 vs 75.5, p = 0.038). Oresland Score (OS) and Pouch Function Scores (PFS) were used to assess patient functional outcomes. Both scores improved in each group over time, converging towards similar results at 12 months. Ta-IPAA scored better on these measures at 12 months (OS – Ta-IPAA 4.6 vs abd-IPAA 6.2, p = 0.02, PFS – Ta-IPAA 6.1 vs abd-IPAA 7.4, p = 0.32).

## Discussion

The 13 papers identified, which describe Ta-IPAA across 9 countries and 3 continents, demonstrate that Ta-IPAA has been adopted across a wide variety of centres, with significant variation in practice regarding the number of operative stages, approach to rectal resection (CRD vs TME), and extraction site. By reviewing and synthesizing the published literature to-date, this analyse provides an overview of current technical nuisances and outcomes in Ta-IPAA and provides a limited comparison against abd-IPAA demonstrating similar post operative outcomes with reduced length of stay.

Several aspects of the operative approach have been hotly debated and examined since its introduction. The stapled J-pouch is now most common due to its simplicity of construction and reliable emptying, whereas the S-pouch is typically reserved for cases where reach is a challenge [48, 49]. Similarly, the stapled ileoanal anastomosis is widely preferred to handsewn with mucosectomy owing to better short-term functional results, reduced rate of stricturing, and ease of creation, except in cases of dysplasia, cancer, or extraintestinal manifestations when complete excision of the rectal mucosa is advised [50–52]. Other aspects such as number of stages, avoidance of defunctioning ileostomy, and plane of dissection remain contested. The single-stage operation is rarely used, while the choice of 2-stage, modified 2-stage, and 3-stage approach is influenced by patient disease processes, physiology, and status of medical management but varies significantly internationally with comparative data primarily from retrospective cohort studies at expert centres [53–57]. Similarly, CRD is preferred in mainland Europe, whereas TME is preferred in the United Kingdom and North America. CRD has been suggested as a means of reducing the risk of nerve and urethral injury and providing a mesorectal “cushion” around the pouch which may help contain small posterior perforations/leaks, but many colorectal surgeons are more accustomed to the TME approach, due to its ubiquity in surgical oncology [58–60]. These international trends are reflected in this pooled analysis. All studies use a J-pouch and only one institution preferred a handsewn anastomosis over stapled. There was varied practice in staging, with a modified 2-stage or 3-stage approach preferred over 1-stage or traditional 2-stage.

While minimally invasive surgery (MIS) is preferable to open surgery, due to the improved female fecundity, reduced adhesions, reduced length of stay and comparable post operative and functional outcomes [7, 61–66], there is still a role for open surgery in this technically demanding procedure, particularly after prior open colectomy due to the increased adhesions, and for resection and transection of the distal rectum and construction of the stapled anastomosis. Many surgeons opt to cross-staple the distal rectum and perform the anastomosis through a small lower midline or pfannenstiel incision when performing MIS. The increased dexterity of robotic instruments may reduce the need for this, and early robotic case series from expert centres demonstrate comparable outcomes to laparoscopic techniques [67–70].

In contrast to transabdominal MIS approaches, transanal MIS provides an alternative approach to the narrow pelvis which in expert hands may overcome the challenges of the abdominal approach, without necessitating conversion to open surgery. Furthermore, the rectum does not need to be cross-stapled as is standard for the double-stapled anastomosis. Instead, the rectal lumen is closed with a purse-string suture and a full thickness rectotomy performed distal to this under direct laparoscopic vision [20]. The specimen can subsequently be extracted through the rectal cuff and anus. Data from RCT and retrospective case matched studies demonstrate reduced post operative patient analgesia requirements and LoS after natural orifice specimen extraction in laparoscopic colectomy and anterior resection [15, 71, 72]. Finally, a single circular stapler fire is used to form the pouch-anal anastomosis, avoiding the need for crossing staple lines. The studies included in this analysis exploited these attributes to varying degrees. Only 3 used transanal extraction preferentially and 2 favoured handsewn anastomosis over single-stapled. It was not possible to perform comparative subgroup analysis examining site of extraction or type of anastomosis due to primary data limitations.

On comparative meta-analysis, anastomotic leak rates between the transanal and transabdominal patient cohorts were comparable. The definition of anastomotic leak varied between papers, but broadly conformed to the principals set out by Rahbari et al., namely disruption of the intestinal wall integrity at the anastomosis with evidence of intra and extraluminal communication, diagnosed by radiological, luminal, or operative findings[24]. Differences in the categorisation of perianastomotic pelvic abscesses without evidence of intraluminal communication, and the diagnostic modalities used between papers may account for the heterogeneity in leak rates. Similarly to our findings, a prior meta-analysis focused on the applicability of Ta-IPAA in the paediatric setting showed outcomes comparable to abd-IPAA (anastomotic leak rate 7.1%, odds ratio 1.36; 95% CI 0.46–4.06, I^2^ = 68%) however a statistically significant difference between LoS was not demonstrated (mean LoS 7.4 days, 95% CI 6–8.8, odds ratio 0.61 days; 95% CI 2.39–1.17) [73]. Functional outcomes after Ta-IPAA appear to be comparable with a transabdominal approach, in keeping with a narrative review of papers specifically investigating functional outcomes after Ta-IPAA [74] but was limited to 1 year follow-up or less.

The limitations of these analyses are the heterogeneity of studies included and the lack of RCT and case-matched series which introduces selection bias. Much of this heterogeneity is a consequence of the broader variation in approaches to pouch surgery – most particularly in staging, abdominal approach, and plane of dissection. The results, though promising, should be interpreted in this context. The systematic review provided here gives a comprehensive overview of current practices and early outcomes from expert centres, which are comparable to those for transabdominal MIS series (LoS 4–14.3 days, post operative morbidity 25–50%, conversion 1.4–13%) [7]. Furthermore, it highlights operative facets which will need to be standardised for high quality RCT comparing transanal and transabdominal approaches. A head-to-head comparison of ta-IPAA with two-teams, transanal extraction, and single-stapled anastomosis against laparoscopic-IPAA with transabdominal extraction and double-stapled anastomosis in a modified 2-stage or 3-stage approach would be valuable and generalisable. This study design would assess the theoretical advantages of the transanal approach while controlling for other confounding operative variables.

It is well recognised that transanal rectal resection has a significant learning curve [75]. Many of the centres captured in this meta-analysis had well established transanal programmes in place prior to adoption of the transanal IPAA technique, and others have been clear about the improvements in their outcomes over time after adaptation. Subgroup analysis from one centre demonstrated a reduction in anastomotic leak rates between cohort first, second and third tertiles (14% vs 14% vs 5%), suggesting that outcomes improved as familiarity with the approach increased [45]. Transanal programmes for rectal cancer resection have been implemented in many centres, which have resulted in improved outcomes for TaTME compared to early attempts at implementation [76, 77]. It would be reasonable to expect that similar gains could be seen by adaptation of the transanal approach to benign disease.

## Conclusion

This review demonstrates the safety, feasibility and reassuring clinical outcomes of Ta-IPAA. Comparative meta-analysis, demonstrates a reduction in length of stay and a trend towards reduced conversion rates compared to abd-IPAA, but was limited by study heterogeneity and a lack of RCTs and case-matched studies, resulting in low to very low certainty of evidence. Robustly designed randomised controlled trials are required to further compare short- and long-term clinical outcomes, as well as quality of life measures. Such trials should take place at centres with established transanal surgery programmes to account for the learning curve associated with these techniques.

### Supplementary Information

Below is the link to the electronic supplementary material.

Supplementary file1 (XLSX 12 KB)
